# Anatomical Characteristics of Cutaneous Branches Extending From the Second Dorsal Metacarpal Artery

**DOI:** 10.3389/fbioe.2020.00995

**Published:** 2020-08-20

**Authors:** Peng Liu, Zhongyuan Deng, Tao Zhang, Xiaojian Li

**Affiliations:** Department of Burn and Plastic, Guangzhou Red Cross Hospital, Medical College, Jinan University, Guangzhou, China

**Keywords:** second dorsal metacarpal artery, cutaneous branches, cluster distribution, skin wound repair, anatomical characteristics

## Abstract

**Background:**

A second dorsal metacarpal artery cutaneous branches flap is often used to repair skin defects in the hand. The location of the cutaneous branch of that artery is very critical for the removal of the flap. In this study, we quantitatively analyzed the origin of the cutaneous branches of the second dorsal metacarpalartery and the distribution characteristics of the radial and ulnar side to provide an anatomical basis for designing a flap.

**Methods:**

Sixteen upper limb specimens were perfused with latex. Four specimens were infused with ethyl acetate plus plastic, and four specimens were perfused with red latex to create pellucid specimens. The origin, travel paths, and distribution of the cutaneous branches of the second dorsal metacarpal artery were anatomically observed, and we measured the length of the cutaneous branch from the midpoint of the second web space edge. We also measured the diameters and pedicle lengths of the radial and ulnar distributions of cutaneous branches of the second dorsal metacarpal artery.

**Results:**

The cutaneous branches of the second dorsal metacarpal artery were mainly clustered at three positions, the second cluster point was at 43.9%, the fourth cluster point was at 61.2%, and the fifth cluster point was at 72.1%. The first cluster point was at 30.8% and the sixth cluster point was at 85.6%. The diameter and pedicle length of the sixth cluster point were the largest. There was no significant difference in the distribution of the diameters and pedicle lengths of the cutaneous branch between the radial and ulnar side. The second dorsal metacarpal artery sent out 1–2 cutaneous branches before the tendon joint, and formed a blood vessel anastomosis with other cutaneous branches located further from the tendon joint. The dorsal branch of the radial nerve in the hand extended a nerve branch at the wrist joint and traveled between the cutaneous branches of the second dorsal metacarpal artery to dominate the corresponding skin.

**Conclusion:**

Three clusters in the distal second dorsal metacarpal artery were selected to be the flap pedicle containing a cutaneous nerve for use in repairing a skin defect in the hand and fingers.

## Introduction

The hand is the most commonly used appendage in daily life and the most vulnerable part of the body. An injured hand is likely to cause physical dysfunction, adversely affect a person’s appearance, and produce apsychological burden (Masakatsu et al., 2018; Viktor and Max, 2018; Xu et al., 2018). Dorsal metacarpal artery flaps are used to repair hand tissue defects, and especially defects of the fingers. Some advantages of the dorsal metacarpal artery flap include a simple operation, convenient tissue transfer, and similarities in characteristics of tissue cortex, toughness, and elasticity (Isaraj, 2011; Schiefer et al., 2012).

The second dorsal metacarpal artery is relatively anatomically consistent and rarely absent. Therefore, a second dorsal metacarpal artery flap is usually used to cover a hand skin defect. Current research shows that the second dorsal metacarpal skin flap is usually designed so as to allow the second metacarpal dorsal artery to serve as the vascular pedicle when repairing small area skin defects in the hand. However, a disadvantage of that design is that it sacrifices the second dorsal metacarpal artery and injures a large amount of tissue (Wang et al., 2011; Chi et al., 2018; Webster and Saint-Cyr, 2020).

Recent studies have shown that the second dorsal metacarpal artery extends cutaneous branches that interconnect in the superficial fascia to form a rich reticular structure rich in blood vessels. The cutaneous branches arising from the dorsal metacarpal artery are mainly distributed in the distal 1/3 segment, and have a mean diameter > 0.2 mm. A cutaneous branch of the second dorsal metacarpal artery can be used as the vascular pedicle when repairing a small area defect in hand (Da-Ping and Morris, 2001; Guang-Rong et al., 2005; Zhang et al., 2009; Appleton and Morris, 2014). However, it is difficult to use ultrasound to verify the exact positions of the cutaneous branches before surgery due to their lack of distinction. Therefore, a quantitative analysis of the anatomical distribution of cutaneous branches is helpful for designing the flap.

Vascular perfusion is a common method to study vascular construction such as blood vessel traveling, distribution, and anastomosis. Different fillers can be used to perfuse blood vessels, and then the blood vessel travel can be displayed by anatomy, transparency, corrosion and radiography. These fillers are rubber, plastic, gelatin, and oil, etc. Latex is the emulsion before the rubber solidifies. The blood vessel specimens perfused with the red latex are elastic, easy to stretch and not easy to break. This method is suitable for microanatomy observation research. Ethyl acetate and plastic perfusion is a method to make cast specimens. The blood vessels are perfused with ethyl acetate and plastic mixed with staining agent. After ethyl acetate and plastic hardening, the specimens are corroded with acid to leave only the ethyl acetate and plastic model of blood vessels. Compared with the latex perfusion, the cast specimens made of ethyl acetate and plastic perfusion can show three-dimensional blood vessels traveling and distribution.

Although numerous studies have described using cutaneous branches flaps with the dorsal metacarpal artery serving as a pedicle, no quantitative analysis has been performed on the distribution patterns of the cutaneous branches, including their radial and ulnar distributions. This study used anatomical techniques such as vascular perfusion, casting, and transparency to study the distribution patterns of the cutaneous branches, including their radial and ulnar distributions, to provide ananatomical basis for designing a flap.

## Materials and Methods

A total of 24 upper limb specimens were legally obtained from the Human Anatomy Department of Southern Medical University in Guangzhou, China. 24 upper limb specimens were amputated at the human elbow joint and immediately the brachial artery was perfused with colored materials. These specimens were placed in a −18°C refrigerator for storage. We performed the anatomical experiments after 1 week. Next, 16 of the specimens were injected with latex for microanatomy examination, four specimens were embedded with ethyl acetate and plastic for use as cast specimens, and four specimens were injected with latex to create transparent specimens. The study protocol was approved by the Institutional Review Board of Guangzhou Red Cross Hospital.

### Latex Specimens for Microanatomy

A glass catheter was carefully inserted into the brachial artery, which was filled with a certain amount of red latex. Next, a longitudinal incision was made between the second and third metacarpal bones on the dorsum of the hand, and the skin tissue was elevated from the deep fascia to expose cutaneous branches extending from the second dorsal metacarpal artery. The lengths, diameters, and positions of cutaneous branches extending from the second dorsal metacarpal artery were measured. The distance between the midpoint of the second web space edge and the midpoint of the second metacarpal bone was set as a unit, and we measured the distance of all branches to the midpoint of the second web space edge.

### Cast Specimens

A glass catheter was carefully inserted into the brachial artery, which was then injected with 10 mL of an ethyl acetate and plastic solution enough to fill the blood vessel; the solution was replenished with a certain amount of ethyl acetate and plastic mixture every 2 h. The mixture was replenished five times in total. The brachial artery was filled with self-setting dental tray material during the final replenishment. After its preparation, the casting specimen was immersed in a 25% hydrochloric acid bath and allowed to slowly corrode in a week. The positions, distribution, and anastomotic connections of cutaneous branches extending from the second dorsal metacarpal artery were observed.

### Transparent Specimens for Direct Observation

An appropriate amount of red latex was perfused into the brachial artery. After it solidified in the blood vessels, the specimen was soaked and subsequently fixed in 75% alcohol; after which, it was air-dried in a ventilated location. Finally, the specimen was soaked in glycerol to make it transparent.

### Statistical Analysis

**FIGURE 1:**
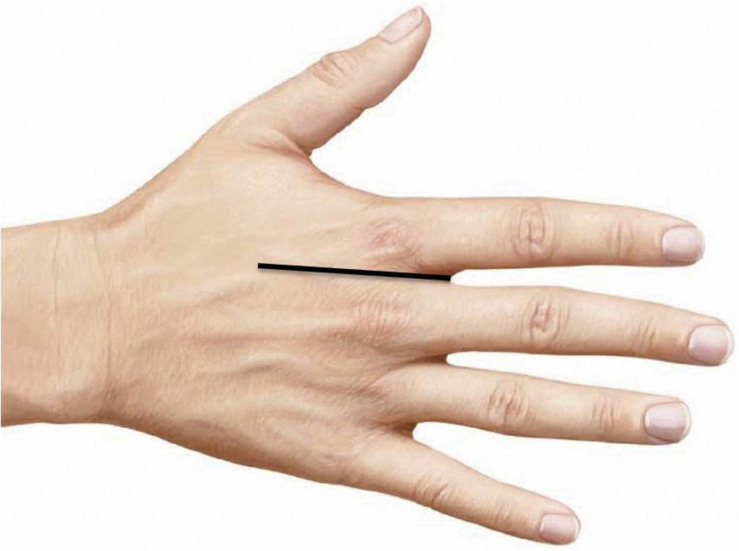
The distance between the midpoint of the second web space edge and the midpoint of the second metacarpal bone was set as the standard unit length (100%).

All data were analyzed using SPSS Statistics for Windows, Version 17.0 (SPSS, Inc., Chicago, IL, United States). The distance between the midpoint of the second web space edge and the midpoint of the second metacarpal bone was established as the standard unit length (100%) (Figure 1). The distance of each cutaneous branch to the midpoint of the second web space edge was recorded. The data were subjected to K-means clustering to quantitatively analyze the origin distribution of the cutaneous branches. The diameters and pedicle lengths of the radial and ulnar distributions of cutaneous branches extending from the second dorsal metacarpal artery were quantitatively analyzed by the independent *t*-test.

## Results

### Origin Distribution of the Cutaneous Branches From the Second Dorsal Metacarpal Artery

**FIGURE 2:**
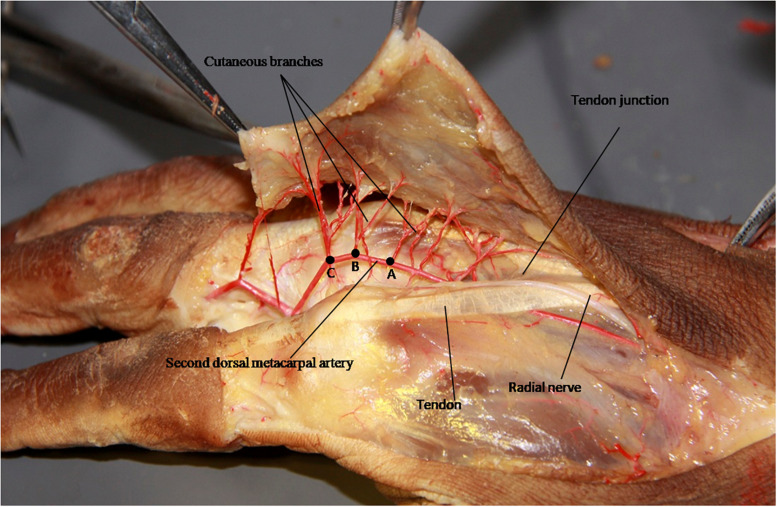
The cutaneous branches from the second dorsal metacarpal artery are mainly clustered at three positions: the second cluster point is at 43.9% **(A)**, the fourth cluster point is at 61.2% **(B)**, and the fifth cluster point was at 72.1% **(C)**.

All cutaneous branches extending from the second dorsal metacarpal artery were counted in 16 specimens, and a total of 103 branches were identified. The cutaneous branches were mainly clustered at three positions: the second cluster point was at 43.9%, and included 21 branches, the fourth cluster point was at 61.2%, and included 22 dermal branches, and the fifth cluster point was at 72.1%, and included 22 cutaneous branches. The first cluster point was at 30.8% and the sixth cluster point was at 85.6%. It was obvious that the cutaneous branches were less distributed at the second cluster point; however, the diameters and pedicle lengths of the branches at the sixth cluster point were the largest (Table 1 and Figure 2).

**TABLE 1:** The clusters distribution of cutaneous branches from the second dorsal metacarpal artery in 16 specimens.

Cluster	Cutaneous branches	Relative distance (%)*	Diameter (mm)	The length of pedicle (mm)
1	9	30.8	0.38 ± 0.15	5.93 ± 1.08
2	21	43.9	0.45 ± 0.13	5.62 ± 2.02
3	16	53.4	0.43 ± 0.13	5.57 ± 1.13
4	22	61.2	0.41 ± 0.11	6.47 ± 1.68
5	22	72.1	0.41 ± 0.17	6.46 ± 2.01
6	13	85.6	0.47 ± 0.20	7.41 ± 1.86

*The distance between the midpoint of the second web space edge and the midpoint of the second metacarpal bone was set as the standard unit length (100%). The relative distance is equal to the distance from each cutaneous branches to the midpoint of the second web space edge divided by the standard unit length.

### Distribution Characteristics of the Diameters and Pedicle Lengths of the Radial and Ulnar Distributions of Cutaneous Branches Extending From the Second Dorsal Metacarpal Artery

**FIGURE 3:**
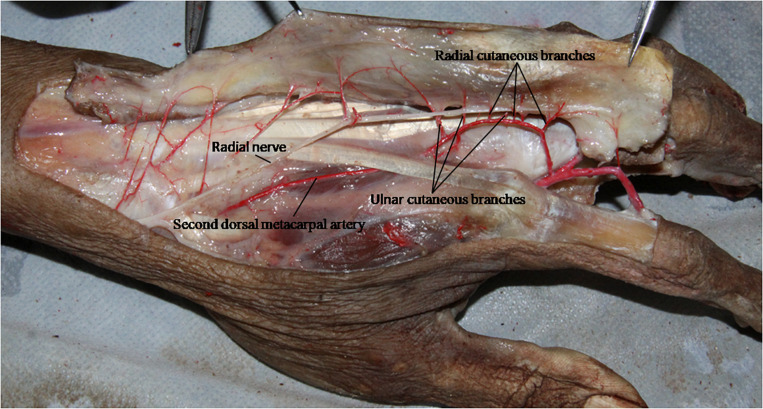
The distribution of the diameter and pedicle length of the cutaneous branch between the radial and ulnar side was no significant difference.

A total of 55 branches were distributed in the radial side of the second dorsal metacarpal artery and 48 branches were distributed in the ulnar side. There were seven more branches in the radial side than in the ulnar side. The mean diameter of the radial branches was smaller than that of the ulnar cutaneous branches; however, there was no significant difference in the distribution of the diameter of the cutaneous branches in the radial and ulnar sides (*p* = 0.659). The mean pedicle length of the radial branches was significantly less than that of the ulnar branches (*p* = 0.265). Therefore, there was no significant difference in the distribution of the diameters and pedicle lengths of the cutaneous branches in the radial and ulnar side (Table 2 and Figure 3).

**TABLE 2:** The distribution of the diameter and pedicle length of the cutaneous branches between the radial and ulnar side.

	Radial (*n* = 55)	Ulnar (*n* = 48)	*t*	*^∗^p*
Diameter (mm)	0.42 ± 0.12	0.43 ± 0.17	−0.443	0.659
The length of pedicle (mm)	6.04 ± 1.64	6.44 ± 1.97	−1.121	0.265

*^∗^*p* was less than 0.05 means that it was significant difference in the distribution of the diameter and pedicle length of the cutaneous branch between the radial and ulnar side.*

### The Anatomical Relationship Between Cutaneous Branches and the Dorsal Branches of the Radial Nerve

**FIGURE 4:**
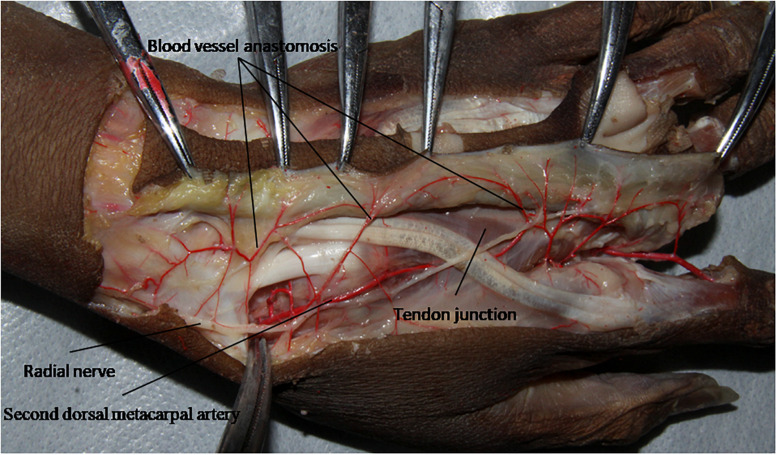
The cutaneous branches extended in the proximal part of the tendon joint, and formed a blood vessel anastomosis with the cutaneous branches farther from the tendon joint.

**FIGURE 5:**
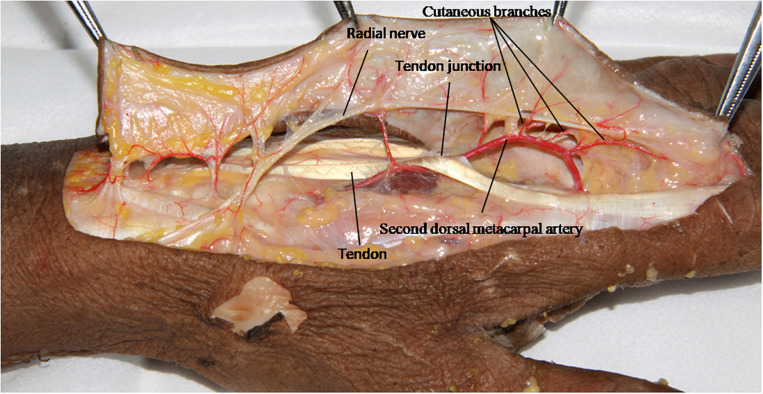
The dorsal branch of the radial nerve in the hand extended a nerve branch at the wrist joint and traveled between the cutaneous branches of the second dorsal metacarpal artery.

The second dorsal metacarpal artery travels between the second and third metacarpal bones, and emits numerous cutaneous branches along the way. The cutaneous branches are mainly concentrated in the distal parts of the second and third tendon joints. However, this anatomical study found that the second dorsal metacarpal artery also emitted 1–2 cutaneous branches prior to the tendon joint, and formed a blood vessel anastomosis with the cutaneous branches located further from the tendon joint. The diameter of the cutaneous branches ranged from 0.31 to 0.47 mm (Figure 4). The dorsal branch of the radial nerve in the hand extended a nerve branch at the wrist joint and traveled between the cutaneous branches of the second dorsal metacarpal artery to dominate the corresponding skin. This anatomical feature can provide an anatomical basis for designing the second dorsal metacarpal artery flap with a sensory nerve (Figure 5).

## Discussion

The second dorsal metacarpal artery flap is an important flap commonly used to repair skin defects in the hand. Cutaneous branches of the dorsal metacarpal artery form a vascular chain that supplies blood for the second dorsal metacarpal artery flap. In this study, we analyzed the origin distribution of cutaneous branches and the diameters and pedicle lengths of the radial and ulnar distribution of cutaneous branches extending from the second dorsal metacarpal artery. The three locations of the clustered cutaneous branches were found to be used for clinicians to design cutaneous branches flap and perform operation. The anatomical adjacent relationship between the cutaneous branches and dorsal cutaneous nerve was also observed.

The second dorsal metacarpal artery originates from the radial artery or dorsal carpal artery network. It then travels on the superficial surface of the dorsal interosseous muscles, and emits numerous cutaneous branches along the way that nourish the corresponding skin tissue (Marx et al., 2001; De Rezende et al., 2004; Al-Baz et al., 2019). Our study found that the cutaneous branches of the second dorsal metacarpal artery were mainly distributed in six clusters, of which there were more cutaneous branches distributed at 43.9, 61.2, and 72.1% of the cluster points. A clinician can locate the vascular pedicle prior to surgery in this position. Our statistical analysis showed that an average of 6.4 branches originated from the second dorsal metacarpal artery. Therefore, we conducted a k-mean clustering analysis to establish six categories for better evaluating the cluster characteristics of cutaneous branches, and obtain more information than could be provided by a two-step cluster analysis (Liu et al., 2015).

**FIGURE 6:**
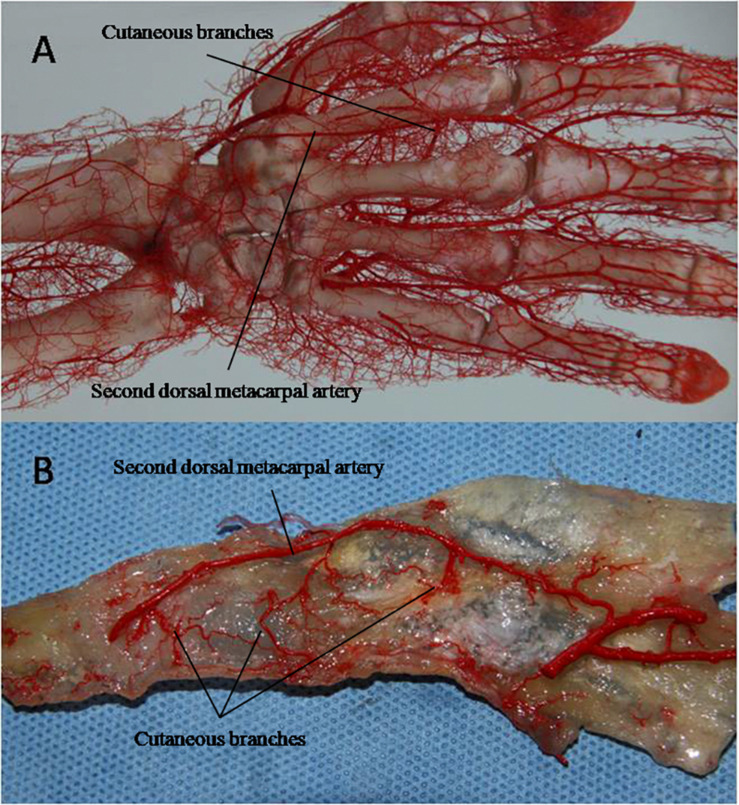
The cast specimens **(A)** and transparent specimens **(B)** also showed no significant difference in the distribution of cutaneous branches on the radius and ulnar side.

Clinically, the second dorsal metacarpal artery flap is designed based on the principle of point, line and surface, and usually has a symmetrical design. However, many vascular branches are often anatomically dominant (Schaverien and Saint-Cyr, 2008; Saint-Cyr et al., 2010; Sun et al., 2013). A study of the radial and ulnar distribution of cutaneous branches is helpful for determining the size and shape of a flap. This study found no significant difference in the distribution of the diameter and pedicle length of the cutaneous branch in the radial and ulnar side (Figure 6).

While most previous studies have focused on the cutaneous branches at the distal part from the tendon joint, there are usually 1–2 cutaneous branches with a diameter of 0.37 ± 0.11 mm before the tendon joint (Yoon et al., 2007; Zhu et al., 2013; Rozen et al., 2015; van Alphen et al., 2016). The cutaneous branches of the distal and proximal parts of the second dorsal metacarpal artery link with each other, which can increase the length of the vascular pedicle of the flap and enlarge its rotational coverage. The dorsal branch of the radial nerve travels between the radial and ulnar cutaneous branches. This anatomical feature can help clinicians to design the second dorsal metacarpal artery flap to include sensory nerves that restore the sensory function of the wound surface and improve the tactile function of the fingertips.

**FIGURE 7:**
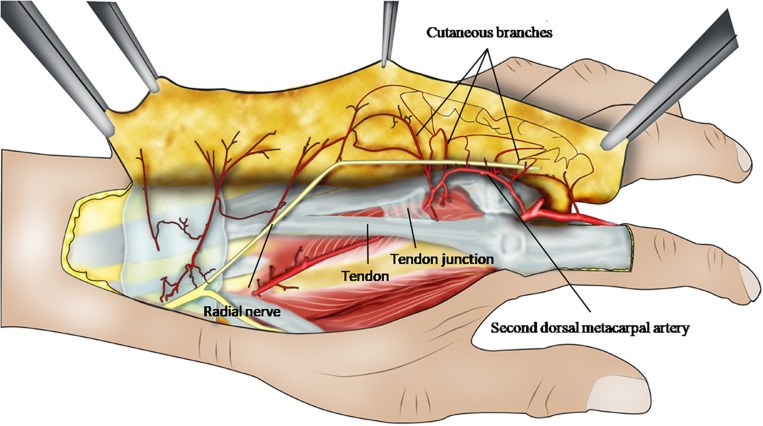
This ideograph presents the anatomical angioarchitecture and the distribution among the cutaneous branches arising from the second dorsal metacarpal artery, and reveals the design of cutaneous branches flap with the second dorsal metacarpal artery pedicle.

**FIGURE 8:**
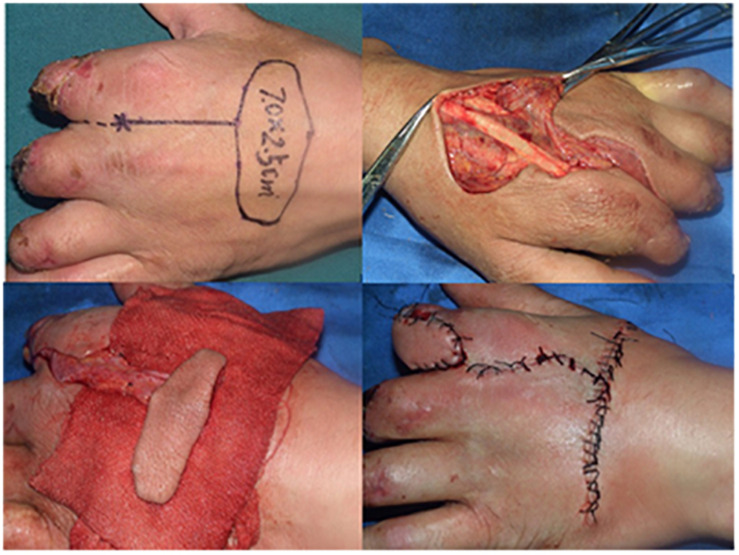
The second dorsal metacarpal artery flap with cutaneous nerve was recovered to repair the skin defect of the hand. After the operation, the patient’s finger movement was normal and finger sensory was good due to 2 mm two-point discrimination.

Based on our anatomical observations and statistical studies, the cutaneous branches near or in the cluster points of the second dorsal metacarpal artery were used as the flap pedicle, and the surface projection of the second dorsal metacarpal artery served as the flap axis. A cutaneous branches flap is designed to cut the plane between the shallow and deep fascia (Figure 7). During the surgical procedure, we preserved the fascial tissue around the pedicle as much as possible in order to avoid vascular spasm caused by excessive distortion or rotation of the cutaneous branches. During the process of lifting the flap, it was not necessary to cut the deep fascia so as to protect both the second dorsal metacarpal artery and the original root of the cutaneous branches. After the operation, the patient’s index finger and middle finger movement was normal and finger sensory function was good, as judged by a 2 mm two-point discrimination (Delikonstantinou et al., 2011; Figure 8).

However, the diameters of cutaneous branches are small enough that naked branches extremely are prone to vasospasm. Before the operation it is very important to perform a Doppler exploration. During the operation, it is necessary to pay attention to the anastomosis of cutaneous branches, and to protect the anastomosis linking between the cutaneous branches as much as possible.

## Conclusion

Cutaneous branches of the second dorsal metacarpal artery were mainly clustered at three positions: 43.9, 61.2, and 72.1% in the distal second dorsal metacarpal artery, which was chosen as the flap pedicle with a cutaneous nerve to repair the skin defect in the hand and fingers of a patient.

## Data Availability Statement

All datasets generated for this study are included in the article/supplementary material.

## Ethics Statement

The studies involving human participants were reviewed and approved by the Institutional Review Board of Guangzhou Red Cross Hospital. Written informed consent obtained from the next of kin.

## Author Contributions

In the beginning of work, XL gave the idea to observe the anastomosis relationship and the distribution of the perforators arising from the second dorsal metacarpal artery. TZ, ZD, and PL dissected carefully the second dorsal metacarpal artery and its perforators, included the linking among the perforators in the superficial fascia and dermis. TZ made the pellucid specimen reveal the course of the perforators and anastomosis between the adjacent branches. In the same time, we measured the diameter and the length of pedicle of the perforators in the course of dissection, then ZD and PL conducted not only a chi-square test to compare the quantities of ulnar and radial branches from second dorsal metacarpal artery in two groups with SPSS 17.0, but also the cluster analysis which was a two-step clustering procedure to observe the integrated distribution of the perforators. ZD and PL design the flap to cover the defect of finger based on the anatomy of second dorsal metacarpal artery perforators. In the whole course, XL provided a great helping for us to complete this research. All authors contributed to the article and approved the submitted version.

## Conflict of Interest

The authors declare that the research was conducted in the absence of any commercial or financial relationships that could be construed as a potential conflict of interest.
